# Morphea Induced by Golimumab in a Patient With Ankylosing Spondylitis: A Case Report and Literature Review

**DOI:** 10.1002/ccr3.72622

**Published:** 2026-04-23

**Authors:** Faten Hilwanjee, Yumn Alghabra, Raghad Aldalati, Nada Al Chama

**Affiliations:** ^1^ Department of Rheumatology Ibn Al‐Nafees Hospital Damascus Syria

**Keywords:** ankylosing spondylitis, anti‐TNFα agents, drug‐related side effects and adverse reactions, golimumab, morphea

## Abstract

Morphea is a localized autoimmune fibrosing disorder that may paradoxically develop during anti‐TNFα agents in ankylosing spondylitis (AS). We report a second case of morphea associated with long‐term golimumab use in a patient with AS, presenting with indurated plaques and confirmed histopathologically. This case highlights the importance of clinical vigilance and timely dermatologic assessment to determine whether morphea represents a drug‐induced reaction or an extra‐articular manifestation of AS.

## Key Clinical Message

Paradoxical morphea may arise during long‐term anti‐TNFα agents in ankylosing spondylitis, reflecting either drug‐induced autoimmunity or an extra‐articular disease feature. Clinicians should promptly evaluate new sclerotic skin lesions to distinguish therapy‐related effects from intrinsic pathogenesis.

## 1 Introduction

Morphea, also known as localized scleroderma, is an autoimmune connective tissue disorder characterized by excessive collagen deposition, resulting in circumscribed skin thickening and fibrosis in the absence of systemic involvement [1]. Its clinical subtypes—plaque, linear, deep, generalized, and mixed—are associated with varying degrees of functional and cosmetic impact [1]. Unlike systemic sclerosis, morphea is characterized by the absence of features such as Raynaud's phenomenon, joint involvement, sclerodactyly, and constitutional symptoms [2]. Although the precise etiology remains unclear, emerging evidence suggests that immune dysregulation, vascular dysfunction, genetic predisposition, and environmental or pharmacological factors may be involved in its pathogenesis [1]. Anti‐TNFα agents are widely used to control autoimmune inflammation, but paradoxical reactions such as morphea have been increasingly reported. Several anti‐TNFα agents have been implicated in these reactions, including adalimumab, etanercept, and infliximab [3]. To the best of our knowledge, only one prior case has specifically linked golimumab to morphea [4]. The management of these reactions typically involves the administration of corticosteroids, methotrexate, and phototherapy [1]. In this report, we present a rare case of morphea occurring in a patient treated with golimumab for ankylosing spondylitis (AS). To emphasize the uniqueness of this case, we reviewed the literature for all reported instances of morphea in patients diagnosed with AS. We aimed to establish whether this manifestation is solely due to anti‐TNFα agents or whether AS itself contributes to the underlying pathogenic mechanism.

## 2 Case History/Examination

**FIGURE 1:**
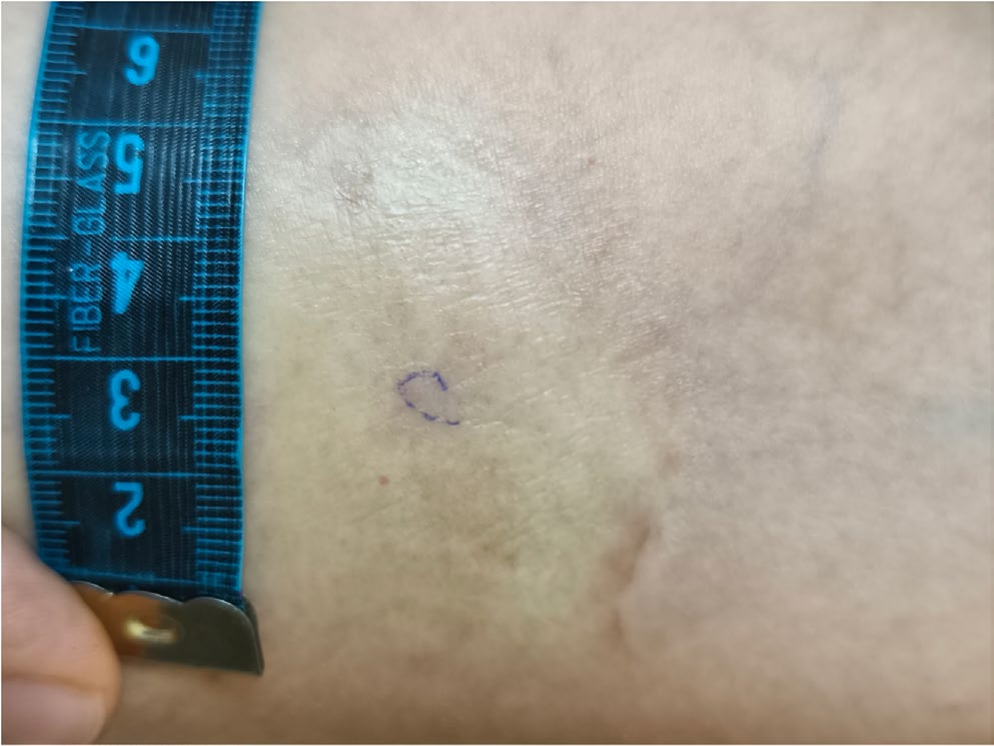
Clinical photograph reveals a solitary, hypopigmented plaque with mild atrophy located on the upper anterior surface of the forearm. The lesion measures approximately 5 × 2 cm. The surface appears smooth and shiny, with subtle dermal atrophy. These features are suggestive of localized morphea.

A 55‐year‐old woman with a history of chronic tobacco use and hypertension was diagnosed seven years ago with HLA‐B27‐positive AS, presenting with inflammatory low back pain and morning stiffness lasting 30 min. Pelvic radiography revealed grade III bilateral sacroiliitis. She was started on monthly subcutaneous golimumab (50 mg) after an inadequate response to NSAIDs. After four years of well‐tolerated therapy, routine examination revealed a violaceous, asymptomatic plaque (5 × 2 cm) on the right forearm, distant from injection sites (Figure 1). Additional firm, partly hypopigmented plaques were noted on the abdomen, axilla, and buttocks. The lesions were neither painful nor pruritic. There was no family history of spondyloarthropathy or morphea.

## 3 Methods (Differential Diagnosis, Investigations and Treatment)

**FIGURE 2:**
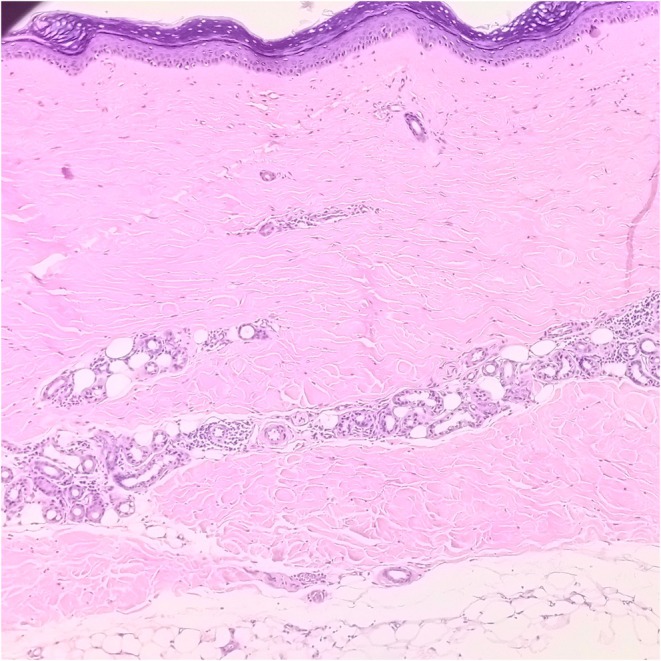
Microscopic examination of the skin biopsy demonstrates a mildly thinned epidermis and a densely collagenized dermis. These histopathological findings are consistent with a diagnosis of scleroderma, reflecting dermal fibrosis and epidermal atrophy characteristic of the condition.

Differential diagnoses included drug‐induced morphea, idiopathic morphea, and systemic sclerosis. A skin biopsy demonstrated dermal sclerosis with dense collagen deposition and loss of adnexal structures, consistent with morphea (Figure 2). Serological tests for systemic sclerosis—including ANA, anti‐centromere, and anti‐Scl‐70 antibodies—were negative. Chest CT ruled out systemic involvement. Based on clinical, histological, and temporal associations, a diagnosis of morphea presumably induced by golimumab was made. Golimumab was discontinued, and high‐potency topical corticosteroids were initiated.

## 4 Conclusions and Results (Outcome and Follow‐Up)

Following the discontinuation of golimumab and the initiation of high‐potency topical corticosteroids, clinical improvement was observed within one month. The progression of the lesions halted, followed by a significant reduction in induration and inflammatory erythema. By the 2‐month follow‐up, the lesions showed approximately 50% resolution with significant softening of the skin, leaving only residual post‐inflammatory hyperpigmentation without signs of active disease. The patient was referred for regular dermatologic follow‐up. This case underscores the importance of recognizing paradoxical cutaneous autoimmunity during anti‐TNFα agents and highlights morphea as a potential delayed‐onset adverse event in AS. Clinicians should maintain vigilance for new or evolving sclerotic skin lesions and pursue timely dermatological evaluation with histopathological confirmation when indicated. Early detection and appropriate management may prevent unnecessary discontinuation of effective therapies. Further case reports and long‐term studies are needed to determine whether morphea represents a treatment‐related side effect, an incidental dermatosis, or an extra‐articular autoimmune manifestation of AS.

## 5 Discussion

Morphea occurring during long‐term golimumab treatment for AS is rare and not well documented. Although anti‐TNFα agents are generally safe and effective, they can occasionally trigger unexpected autoimmune skin reactions, including psoriasis, lupus‐like rashes, and morphea [5, 6, 7]. The higher number of morphea cases reported with adalimumab may reflect its broader clinical use compared to other anti‐TNFα agents [8]. In contrast, morphea associated with golimumab is extremely rare.

In our patient, sclerotic skin plaques appeared after four years of continuous golimumab therapy—significantly later than the typical 12–24 months reported in literature [4]. This delayed onset suggests a gradual shift in immune regulation rather than an acute hypersensitivity reaction. The distribution of firm plaques across multiple body areas, in the absence of systemic symptoms or abnormal laboratory findings, supports a diagnosis of localized scleroderma and points toward a drug‐related etiology.

TNF‐α plays a central role in inflammation but also helps prevent fibrosis by limiting fibroblast proliferation and collagen synthesis [9]. Its inhibition may lead to increased activity of transforming growth factor‐beta (TGF‐β), which promotes fibroblast activation and extracellular matrix accumulation, contributing to skin thickening and fibrosis as seen in morphea [8].

A review of three previously reported cases of AS‐associated morphea (Table 1)—two during anti‐TNFα agents and one in an untreated patient—suggests two possible mechanisms. First, anti‐TNFα agents may trigger paradoxical immune phenomena resulting in cutaneous autoimmunity [7]. Second, morphea and AS may share overlapping immunopathogenic pathways, particularly Th17 dysregulation. Both conditions involve activation of Th1, Th2, and Th17 cells, with key cytokines such as IL‐17 and IL‐23 playing prominent roles. This immunological overlap raises the possibility that morphea may represent a rare extra‐articular manifestation of AS [10, 11].

**TABLE 1:** Summary of reported cases of morphea in patients with ankylosing spondylitis, with or without anti‐TNFα therapy.

Author (year)	Age	Sex	Anti‐TNF agent	Drug latency (months)	Morphea type/site	Outcome
Ramírez et al. [3]	37	Male	Adalimumab	12	Plaque/two in the lower limbs	The lesion regressed after drug withdrawal, leaving only mild skin hyperpigmentation
Torrente‐Segarra et al. [4]	58	Female	Golimumab	20	Linear/lower limbs	The treatment was not reported
Gümüşsu et al. [2]	60	Female	None	Not applicable	Plaque/one on the shoulder	Responded to topical corticosteroids

In our case, partial improvement of skin lesions following golimumab discontinuation supports the hypothesis of a drug‐induced reaction. Interestingly, the untreated patient had plaque‐type morphea, while those receiving anti‐TNFα agents developed more widespread or linear forms, possibly reflecting differences in immune modulation induced by biologic therapy.

This case emphasizes the need for diagnostic vigilance and therapeutic awareness in evaluating localized sclerotic skin lesions. Clinicians should consider morphea in atypical presentations and pursue early histopathologic confirmation. Its emergence during long‐term anti‐TNFα agents such as golimumab suggests a possible drug‐related mechanism and warrants further research to clarify its link to AS and guide monitoring strategies.

## Author Contributions

**Faten Hilwanjee:** writing – original draft. **Yumn Alghabra:** writing – original draft, writing – review and editing. **Raghad Aldalati:** investigation. **Nada Al Chama:** conceptualization, supervision.

## Funding

The authors have nothing to report.

## Ethics Statement

Not required for single case reports by our local ethics board.

## Consent

Written informed consent was obtained from the patient to publish this report in accordance with the journal's patient consent policy.

## Conflicts of Interest

The authors declare no conflicts of interest.

## Data Availability

All data are included within the article.

## References

[1] C. Papara , D. A. De Luca , K. Bieber , A. Vorobyev , and R. J. Ludwig , “Morphea: The 2023 Update,” Frontiers in Medicine 10 (2023): 1108623. 36860340 10.3389/fmed.2023.1108623 PMC9969991

[2] K. Gümüşsu , A. Rezvani , N. Özaras , and M. Güler , “Coexistence of Ankylosing Spondylitis With Morphea: A Case Report,” Archives of Rheumatology 29, no. 2 (2014): 143–146.

[3] J. Ramírez , M. V. Hernández , J. Galve , J. D. Cañete , and R. Sanmartí , “Morphea Associated With the Use of Adalimumab: A Case Report and Review of the Literature,” Modern Rheumatology 22 (2012): 602–604. 22095405 10.1007/s10165-011-0550-4

[4] V. Torrente‐Segarra , P. Campo , S. Heredia , C. Heras‐Mulero , and M. Bonet , “Linear Localized Morphea Associated With Golimumab in a Patient With Spondyloarthritis,” Reumatologia Clinica 16, no. 4 (2018): 303–305. 30120020 10.1016/j.reuma.2018.06.003

[5] M. V. Hernández , M. Meineri , and R. Sanmartí , “Skin Lesions and Treatment With Tumor Necrosis Factor Alpha Antagonists,” Reumatología Clínica 9, no. 1 (2013): 53–61. 22766431 10.1016/j.reuma.2012.04.007

[6] M. Flendrie , W. H. P. M. Vissers , M. C. W. Creemers , E. M. G. J. de Jong , P. C. M. van de Kerkhof , and P. L. C. M. van Riel , “Dermatological Conditions During TNF‐α‐Blocking Therapy in Patients With Rheumatoid Arthritis: A Prospective Study,” Arthritis Research & Therapy 7 (2005): 1–11. 15899052 10.1186/ar1724 PMC1174960

[7] H. Lee , I. Song , M. Friedrich , et al., “Cutaneous Side‐Effects in Patients With Rheumatic Diseases During Application of Tumour Necrosis Factor‐α Antagonists,” British Journal of Dermatology 156, no. 3 (2007): 486–491. 17300238 10.1111/j.1365-2133.2007.07682.x

[8] K. Maliyar , A. Mufti , M. Sachdeva , Y. Lytvyn , J. Salsberg , and J. Yeung , “Development of Morphea in Patients Receiving Biologic Therapies: A Systematic Review,” Journal of the American Academy of Dermatology 84, no. 4 (2021): 1081–1085, 10.1016/j.jaad.2020.06.1027. 32687966

[9] J. P. Reinhart , J. L. Aird , M. C. Stephens , S. Asch , A. B. Orandi , and M. M. Tollefson , “Tumor Necrosis Factor‐α Inhibitor‐Induced Morphea and Psoriasiform Dermatitis in a Pediatric Patient With Crohn's Disease,” Pediatric Dermatology 40, no. 3 (2023): 519–522. 36385392 10.1111/pde.15182

[10] A. Dańczak‐Pazdrowska , M. Kowalczyk , B. Szramka‐Pawlak , et al., “Interleukin‐17A and Interleukin‐23 in Morphea,” Archives of Medical Science 8, no. 6 (2012): 1089–1095. 23319986 10.5114/aoms.2012.32421 PMC3542501

[11] T. A. Wynn , “Type 2 Cytokines: Mechanisms and Therapeutic Strategies,” Nature Reviews. Immunology 15, no. 5 (2015): 271–282. 10.1038/nri3831 25882242

